# Evaluation of the Pose Tracking Performance of the Azure Kinect and Kinect v2 for Gait Analysis in Comparison with a Gold Standard: A Pilot Study

**DOI:** 10.3390/s20185104

**Published:** 2020-09-08

**Authors:** Justin Amadeus Albert, Victor Owolabi, Arnd Gebel, Clemens Markus Brahms, Urs Granacher, Bert Arnrich

**Affiliations:** 1 Connected Healthcare, Digital Health Center, Hasso Plattner Institute, University of Potsdam, 14482 Potsdam, Germany; Victor.Owolabi@student.hpi.uni-potsdam.de; 2 Division of Training and Movement Sciences, University of Potsdam, 14469 Potsdam, Germany; arnd.gebel@uni-potsdam.de (A.G.); mbrahms@uni-potsdam.de (C.M.B.); urs.granacher@uni-potsdam.de (U.G.)

**Keywords:** motion capture, evaluation, human motion, RGB-D cameras, digital health

## Abstract

Gait analysis is an important tool for the early detection of neurological diseases and for the assessment of risk of falling in elderly people. The availability of low-cost camera hardware on the market today and recent advances in Machine Learning enable a wide range of clinical and health-related applications, such as patient monitoring or exercise recognition at home. In this study, we evaluated the motion tracking performance of the latest generation of the Microsoft Kinect camera, Azure Kinect, compared to its predecessor Kinect v2 in terms of treadmill walking using a gold standard Vicon multi-camera motion capturing system and the 39 marker Plug-in Gait model. Five young and healthy subjects walked on a treadmill at three different velocities while data were recorded simultaneously with all three camera systems. An easy-to-administer camera calibration method developed here was used to spatially align the 3D skeleton data from both Kinect cameras and the Vicon system. With this calibration, the spatial agreement of joint positions between the two Kinect cameras and the reference system was evaluated. In addition, we compared the accuracy of certain spatio-temporal gait parameters, i.e., step length, step time, step width, and stride time calculated from the Kinect data, with the gold standard system. Our results showed that the improved hardware and the motion tracking algorithm of the Azure Kinect camera led to a significantly higher accuracy of the spatial gait parameters than the predecessor Kinect v2, while no significant differences were found between the temporal parameters. Furthermore, we explain in detail how this experimental setup could be used to continuously monitor the progress during gait rehabilitation in older people.

## 1. Introduction

Human gait is an important indicator related to different markers of health (e.g., age-related diseases or early mortality). More specifically, gait speed has been denoted as the sixth vital sense because it is associated with daily function, late-life mobility, independence, falls, fear of falls, fractures, mental health, cognitive function, adverse clinical events, hospitalization, institutionalization, and survival [1]. Accordingly, gait analysis is indispensable for the description of underlying gait patterns, especially in patients with neurological diseases such as Parkinson’s disease [2]. Spatio-temporal parameters help to describe the gait pattern of patients quantitatively and objectively. For instance, reduced step length, prolonged double-stance phases, increased step width can often be found in Parkinsonian patients [3]. An established gold standard for gait analysis are multi-camera motion capturing systems that track retro-reflective markers. Based on the marker positions in the individual camera images, their 3D positions can be reconstructed using triangulation. In general, motion capturing systems, such as the well-known Vicon system (Vicon Motion Systems, Oxford, UK), offer high accuracy [4]. However, these camera systems are expensive and require a large setup in specialized gait analysis laboratories as well as a time-consuming marker placement on the participants. In order to be able to take measurements outside the laboratory, the Microsoft Kinect sensor has been identified as a portable and cost-effective device for gait assessment [5].

The Kinect v1, launched by Microsoft in 2010, has an integrated RGB and an infrared (IR) camera, capable of tracking users in 3D. This sensor was designed as a gaming controller for the Xbox, allowing users to interact with games without the use of a controller device. For depth estimation, the Kinect v1 uses the principle of structured light, in which an IR projector sequentially illuminates the scene with a known and fixed dot pattern. By observing the dot pattern with an IR camera, the depth information can be estimated using triangulation techniques, where the baseline between camera and projector is known [6]. The Artificial Intelligence (AI)-based skeleton tracking uses randomized decision forests trained on a large data set of labeled depth images [7].

The Kinect v2 was launched in 2014, with improved hardware and skeleton tracking. The depth estimation method has changed to the time-of-flight (ToF) principle, in which the distance to an object is determined by the time it takes for the emitted light to reach the object and return to the sensor [8]. The Kinect for Windows SDK 2.0 allows for tracking the 3D positions and orientations of 25 joints of six users simultaneously [9]. However, Microsoft has stopped the production of the Kinect v2 [10].

In 2019, Microsoft launched the Azure Kinect DK (Developer Kit). It can easily be integrated with Azure Cognitive Services, a library of various AI applications, such as speech and image analysis [11]. In a 2019 review, Clark et al. [12] were not able to determine whether the Azure Kinect could be used as a standalone device or only as part of a web platform. However, it turned out, this camera can still be used as a local peripheral device. The Azure Kinect camera also consists of an RGB camera and an IR camera, which utilizes the ToF principle, but offers significantly higher accuracy than other commercially available cameras [13]. In addition, the Kinect camera has an integrated inertial measurement unit (IMU) and a 7-microphone array, extending the range of possible applications to other areas. Microsoft has also developed a new body tracking SDK for the Azure Kinect which is based on Deep Learning (DL) and Convolutional Neural Networks (CNN).

Notably, depth sensors other than the Microsoft Kinect are commercially available, such as the Intel Realsense, Stereolabs ZED, or Orbbec [14]. By using either the manufacturer’s skeleton tracking software solutions or other available human pose estimation methods that also work on 2D RGB images, such as OpenPose [15], DeepPose [16], or VNect [17], these depth cameras also have the potential to be used for healthcare applications. However, according to Clark et al. [12], the accuracy of other depth cameras in comparison to the Kinect cameras has not yet been established.

Against this background, the present study investigates whether the Azure Kinect camera can be used for accurate gait assessment on a treadmill by comparing the collected data to a gold standard (Vicon 3D camera system). We also evaluate whether its improved hardware and DL-based skeleton tracking algorithm performs better than its predecessor, the Kinect v2. The structure of this paper is as follows: Section 2 gives an overview of the related literature and presents applications in which the Kinect v1 and Kinect v2 cameras were evaluated with respect to clinical use cases. Section 3 introduces the hardware used and the methods developed. The evaluation results of the human pose estimation performance and concurrent comparison between both Kinect cameras is presented in Section 4 and concludes with a discussion in Section 5.

## 2. Related Work

In recent years, Kinect v1 and Kinect v2 were extensively studied by the research community to see if these sensors can be used as alternative measurement devices for assessing human motion. Among these studies, several constellations and configurations were used, such as performing physical exercises at a static location, walking along a walkway, or walking on a treadmill. In addition, some studies used multiple Kinect sensors simultaneously and integrated the resulting data into a global coordinate system, resulting in more robust tracking and extended camera range [18]. However, since only a single Kinect sensor was used in this study to evaluate the gait, the focus lies on related literature that studied gait with a single camera.

Wang et al. [19] examined the differences in human pose assessment between Kinect v1 and Kinect v2 in relation to twelve different rehabilitation exercises. They also evaluated the quality changes when filming from three different viewing angles. Ultimately, they concluded that Kinect v2 has overall better accuracy in joint estimation and is also more robust to occlusion and body rotation than Kinect v1. The study by Galna et al. [20] examined the accuracy of the Microsoft Kinect sensor for measuring movement in people with Parkinson’s disease. Movement tasks included quiet standing, multidirectional grasping and various tasks from the Unified Parkinson’s Disease Rating Scale such as hand gripping and finger tapping. These tasks were performed by nine Parkinsonian patients and 10 healthy controls. It was found that Kinect performs well for temporal parameters, but lacks in accuracy for smaller spatial exercises such as toe tapping.

Capecci et al. [21] used the Kinect v2 sensor to evaluate rehabilitation exercises for lower back pain, such as lifting the arms and performing a squat. Angles and joint positions were defined as clinically relevant features and the deviation was analyzed with respect to a reference motion capture system. It was concluded that the Kinect v2 captured the timing of exercises well and provided comparable results for joint angles. Overground walking recorded with Kinect v2 was evaluated by Mentiplay et al. [22] by studying spatio-temporal and kinematic walking conditions. There was excellent relative agreement on the measurement of walking speed, ground contact time, vertical displacement range of the center of the pelvis, foot swing speed as well as step time, length and width at both speeds, comfortable and fast. However, the relative agreement for kinematic parameters was described as being poor to moderate.

Xu et al. [23] investigated whether the Kinect v2 sensor could be used to measure spatio-temporal gait parameters on the treadmill. Using 20 healthy volunteers, they validated gait parameters such as step time, step time, swing phase, stance phase, and double limb support time as well as kinematic angles for walking at three different speeds. Their results showed that gait parameters based solely on heel strikes had lower errors than those based on toe off events. This could be caused by the camera angle, i.e., test subjects were being filmed from the frontal plane, so that the corresponding joints were closer to the camera during heel strike. Macpherson et al. [24] evaluated the tracking quality of a pointcloud-based Kinect system for measuring pelvic and trunk kinematics for treadmill walking. The three-dimensional linear and angular range of motion (ROM) of the pelvis and trunk was evaluated and compared with a 6-camera motion capturing system (Vicon). They obtained very large to perfect within subjects correlation coefficients (*r* = 0.87–1.00) for almost all linear ROMs of the trunk and pelvis. However, the authors report less consistent correlations for the angular ROM, ranging from moderate to large.

## 3. Materials and Methods

This chapter introduces the hardware used and the methods developed in order to compare the human pose estimation performance between both Kinect cameras and the Vicon high resolution motion capturing system.

### 3.1. Microsoft Kinect v2

**Figure 1:**
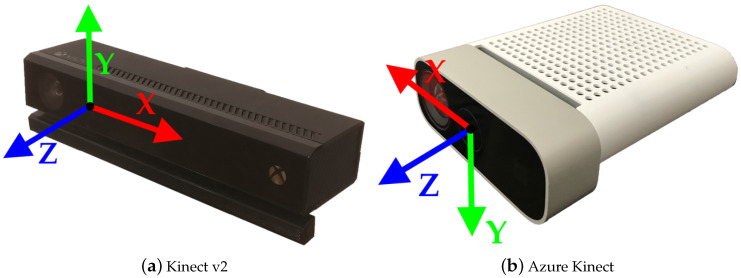
Both Kinect cameras used for this study with depicted camera coordinate systems.

The Microsoft Kinect v2 camera consists of a RGB and an IR camera as well as three IR projectors. The sensor is utilizing the ToF principle for depth estimation. The RGB camera has a resolution of 1920 × 1080 px and the IR camera has a resolution of 512 × 424 px while both cameras deliver frames at 30 Hz. Data from the sensor can be accessed using the Kinect for Windows SDK 2.0, which allows for tracking up to 6 users simultaneously with 25 joints each. For each joint, the three-dimensional position is provided as well as the orientation as quaternion. The center of the IR camera lense represents the origin of the 3D coordinate system, in which the skeletons are also tracked, as shown in Figure 1a. Table 1 provides a more detailed overview of the technical specifications of the Kinect v2 compared to the Azure Kinect camera.

### 3.2. Microsoft Azure Kinect

The Azure Kinect camera also consists of a RGB camera and an IR camera. The color camera offers different resolutions, the highest resolution being 3840 × 2160 px at 30 Hz. The IR camera has a highest resolution of 1 MP, with 1024 × 1024 px. This Kinect sensor also utilizes the ToF principle. In addition, both the RGB and IR cameras support different fields of view. The Azure Kinect also has an IMU sensor, consisting of a triaxial accelerometer and a gyroscope, which can estimate its own position in space. For skeletal tracking, Microsoft offers a Body Tracking SDK, which is available for Windows and Linux and the programming languages C and C++. This SDK is able to track multiple users with 32 joints each. In contrast to the skeleton definition of the former Kinect generation, the current definition includes more joints in the face, such as ears and eyes. As its older generations, the SDK provides the joint orientations in quaternions representing local coordinate systems, as well as three-dimensional position data. The origin of the 3D coordinate system is also the center of the IR camera, but having some axes expand into opposite directions, as presented in Figure 1b.

### 3.3. Experimental Setup and Data Collection

**Figure 2:**
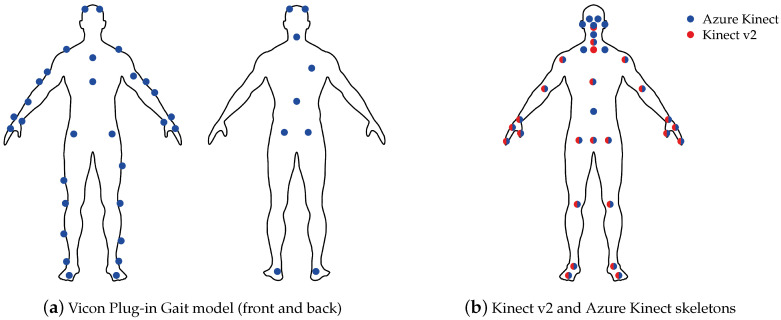
Marker setups for Vicon and both Kinect cameras.

**Figure 3:**
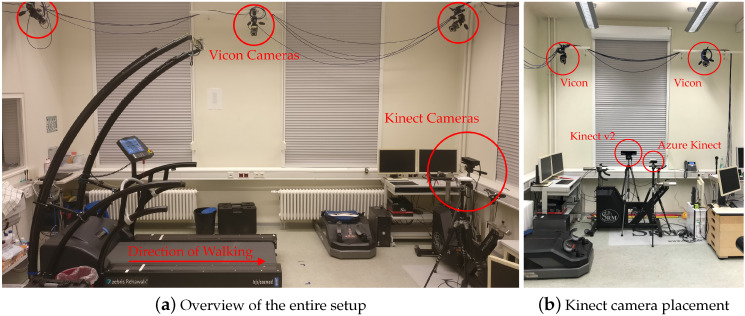
Experimental setup in the laboratory. Both Kinect cameras were placed in front of the treadmill.

This study was approved by the Ethics Committee of the University of Potsdam (Application 29/2020). Before the data collection, informed consent was given by the participants. Five young and healthy adults (average age of 28.4 ± 4.2 years, body mass of 73.4 ± 10.6 kg, and height of 178.8 ± 7.0 cm) walked on a treadmill (h/p/cosmos, Nussdorf-Traunstein, Germany) at three different speeds. The experiments were conducted at the lab of the Division of Training and Movement Science at the University of Potsdam. Speeds were selected according to the study presented by Xu et al. [23], starting at 3 kmh${}^{-1}$≈ 0.85 ms${}^{-1}$ followed by 3.9 kmh${}^{-1}$≈ 1.07 ms${}^{-1}$ and finally 4.7 kmh${}^{-1}$≈ 1.3 ms${}^{-1}$. Each subject was asked to perform each trial of a certain speed twice and to walk 100 steps per side. The participants were equipped with the 39 retro-reflective marker full-body Plug-in Gait model [25] with markers corresponding to anatomical landmarks, as shown in Figure 2a. In addition, the participants walked barefoot, firstly to be able to attach the foot markers precisely and secondly to avoid movement artifacts through the fabric of the shoes. 3D marker kinematics were captured at 100 Hz using ten IR cameras (Vicon, Bonita 10 [26]), as shown in Figure 3a. Vicon Nexus 2.10.1. software was used for data recording and post-processing of the data.

The joints tracked by both Kinect cameras are approximate and anatomically incorrect. As mentioned above, the Kinect v2 and Azure Kinect cameras track 25 joints and 32 joints, respectively. Most joints between the Kinect systems are similar. However, the Azure Kinect model contains additional markers, e.g., for ears, eyes, and clavicles. Figure 2b shows the marker definitions from Kinect v2 and Azure Kinect together.

Both Kinect cameras were placed at a distance of 3–4 meters in front of the treadmill to stay within the recommended range for body tracking. Each camera was mounted on a tripod at approximately one meter above the floor, as shown in Figure 3b. During a pilot experiment, it was found that the Azure Kinect skeleton tracking generated artifacts such as skeleton jitter and frame loss. These artifacts disappeared after the Azure Kinect camera was moved closer to the treadmill and the subject. However, the reason for these initial problems is still unknown. Due to the different distances to the treadmill of both Kinect cameras, the height of the Azure Kinect was slightly lowered (also noticeable in the figure) in order to have aligned visible treadmill areas in both camera images. Each Kinect camera was connected to its own laptop running a customized recording software. The laptop used to run the Azure Kinect was equipped with a Nvidia^TM^ GeForce RTX 2060 GPU with 6 GB of graphics memory, in order to have sufficient power to run the DL model for body tracking, as well as 16 GB of RAM and an Intel^®^ i7-8750H 2.20 GHz CPU. The software for storing the pose and image data of the Azure Kinect was implemented using the programming language C++ and the Body Tracking SDK. At the time this study was conducted, the Body Tracking SDK was available in version 1.0.1.

The Microsoft Kinect v2 camera data was recorded with custom software using the *PyKinect2* library for the Python^TM^ programming language, which is based on the Kinect for Windows 2.0 SDK. The recording laptop had 16 GB of RAM installed and was running with an Intel^®^ i7-8565U 2.20 GHz CPU.

### 3.4. Signal Processing and Synchronization

Data post-processing included the reconstruction of the model and the labeling of the markers. Present gaps in the data were filled manually within the Vicon Nexus software. Data were filtered using a 4th-order Butterworth filter with a cut-off frequency of 5 Hz.

The Kinect v2 camera has a target sampling rate of 30 Hz, but, during the experiments, it was found that the frequency was not constant. This was shown by calculating the time difference between consecutive examples across all experiments, resulting in a sampling rate of 37.73 ± 25.80 ms. Since the Kinect v2 data were not sampled constantly, the signal was resampled at a constant sampling frequency as proposed by Scano et al. [27]. Furthermore, the signal was upsampled to 100 Hz in order to match the Vicon sampling frequency.

Similar to the Kinect v2, the Azure Kinect also provides a sampling frequency of 30 Hz. The calculated differences over all measured frames gave a mean value of 34.23 ± 14.92 ms. However, the recorded time stamps in the data showed that the time differences of the measurements were constant, but some gaps were present. These gaps were identified in the developed software and filled using quadratic interpolation. After filling the gaps, the pose data were also upsampled to 100 Hz to match the Vicon sampling frequency.

**Figure 4:**
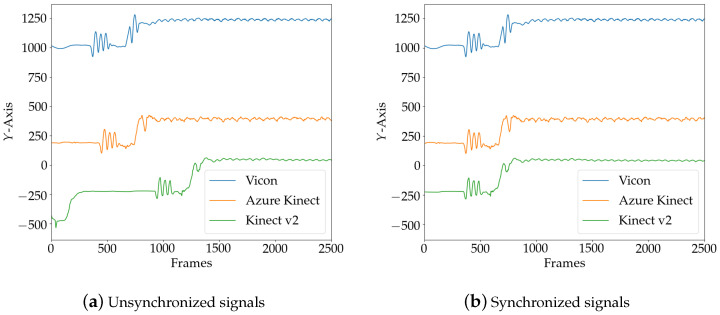
Temporal alignment of Vicon and both Kinect camera signals sampled at 100 Hz. The three consecutive peaks indicate the jumping procedure.

The participants were asked to perform three consecutive jumps before and after each trial to obtain significant peaks in the pose data. During post-processing, the data of both Kinect sensors were synchronized with the Vicon reference system using cross-correlation of the second derivative of a central marker in the vertical axes. Therefore, the Sternum marker (STRN) of the Plug-in Gait model was used, and the Pelvis marker for Kinect v2 and Azure Kinect. Figure 4a shows the unsynchronized signals from the three camera systems, with both Kinect signals upsampled to 100 Hz and the peaks from the jumping protocol visible. The final synchronization result using the cross-correlation shift method is shown in Figure 4b.

### 3.5. Spatial Alignment of Skeleton Data

**Figure 5:**
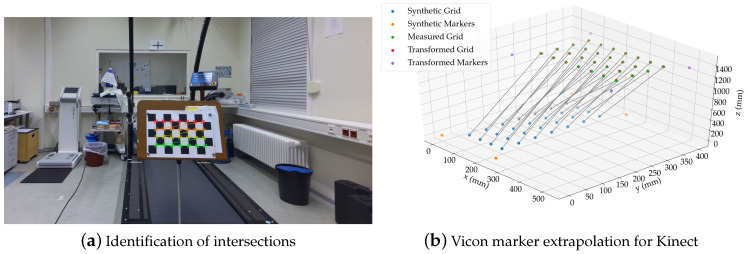
Camera calibration using four retro-reflective 14 mm markers on the corners of the checkerboard.

To calculate the spatial deviation of the two Kinect cameras and the ground truth data from the Vicon system, the kinematic data from all three systems were transformed into a global coordinate system. For both Kinect cameras, the origin of the coordinate system is the center of the IR camera. The *y*-axis of the Kinect v2 camera points upwards, while the same axis points downwards in the Azure Kinect. The *x*-axes also point in the opposite direction along the camera. The *z*-axis extends in the direction of view in both systems (as shown in Figure 1a,b). The Vicon coordinate system was defined by the triangular calibration wand, which was placed parallel to the treadmill. To transform each Kinect coordinate system into the global system, the Vicon system, an 8 × 5 checkerboard pattern with a length of s=50 mm per square side was used. Four retro-reflective 14 mm markers were placed at the outer corners of the checkerboard, as shown in Figure 5a. The goal of the calibration method was to find a transformation to map simultaneously recorded points from one system to another system.

As mentioned by Naeemabadi et al. [28], retro-reflective markers, like those of the Vicon system, appear as noise in the IR images, so their 3D position is unknown to the Kinect sensors. To determine the 3D position of the Vicon markers, nevertheless, the intermediate intersection points on the chessboard were identified in the 2D color images using the OpenCV library, followed by the coordinate mapper functions provided by the Kinect SDKs to obtain the actual 3D positions from the depth camera space, denoted as points $p_{i}$ in the point set *P*. A transformation was then found by fitting a grid with the same dimensions into the point set and using this transformation to extrapolate the known Vicon marker positions in 3D space. The grid *Q* consisting of points $q_{i}$ with known distances was generated as shown in Equation (1), where *h* and *w* denote the dimensions of the used checkerboard:

(1) $$Q=\left\{\left(\begin{array}{c} x\\ y\\ 0 \end{array}\right)\cdot s\in R^{3}:1\leq x\leq w,1\leq y\leq h\right\}$$

To register the two point sets *P* and *Q* with known point correspondences, a rigid transformation must be found, which consists of a rotation matrix $R\in R^{3\times 3}$ and a translation vector $t\in R^{3}$. Therefore, a method based on singular value decomposition (SVD) was used [29]. Since the checkerboard data contained measurement errors, the objective of the algorithm was to find a rigid transformation such that the least squares error in Equation (2) was minimized:

(2) $$\left(R,t\right)=\underset{R\in R^{3\times 3},t\in R^{3}}{arg min}\sum_{i=1}^{n}{∥\left(Rp_{i}+t\right)-q_{i}∥}^{2}$$

To minimize the objective, both point sets of size *n* were moved to their respective origin by subtracting for each point set its centroid $\bar{p}$ and $\bar{q}$ as shown in Formulas (3) and (4):

(3) $$\bar{p}=\frac{1}{n}\sum_{i=0}^{n}p_{i}\;\bar{q}=\frac{1}{n}\sum_{i=0}^{n}q_{i}$$

(4) $$x_{i}:=p_{i}-\bar{p},\;y_{i}:=q_{i}-\bar{q},\;i=1,2,\ldots ,n$$

The next step was to calculate the covariance matrix $S\in R^{3\times 3}$, where *X* and *Y* are matrices that hold the centered vectors $x_{i}$ and $y_{i}$ in their columns, as shown in Equation (5):

(5) $$S=XY^{T}$$

Subsequently, the rotation matrix was obtained by performing a SVD on the covariance matrix $S=U\Sigma V^{T}$. The rotation matrix was then calculated as shown in Equation (6), and the final translation vector was calculated as shown in Formula (7):

(6) $$R=V\left(\begin{array}{ccc} 1 & \; & \\ \; & 1 & \\ \; & \; & det(VU^{T}) \end{array}\right)U^{T}$$

(7) $$t=\bar{q}-R\bar{p}$$

The obtained transformation with *R* and *t* was then applied to the Vicon marker locations of the synthetically generated grid to obtain the real locations with respect to the Kinect coordinate system. Then, the above algorithm from Formulas (2) to (7) was applied to the extrapolated Vicon markers for Kinect and the measured Vicon points *V* to obtain a transformation that maps the Kinect points into the Vicon coordinate system. Throughout the calibration procedure, the checkerboard was moved several times in the area to scan enough calibration points. The calibration algorithm was applied for both Kinect cameras separately.

### 3.6. Gait Parameter Calculation

Since the aim of this study was to evaluate spatio-temporal gait parameters, i.e., step length, step time, step width and stride time, these parameters were calculated based on the method presented by Zeni et al. [30]. The authors proposed an algorithm to automatically detect heel strike and toe off events in kinematic data and to derive swing and stance phases from it. It was shown that this method determines the timing of gait events with an error of 1/60 s compared to a vertical ground reaction force (GRF).

In this method, the anterio-posterior direction (e.g., the *x*-axis) of the foot marker trajectory is plotted over time which results in a sinusoidal curve as the treadmill belt constantly pulls back the foot after every heel strike [30]. Thus, the heel strikes at a given time $t_{HS}$ were determined by finding the maximum peaks within the 1D heel marker signal $x_{heel}$, while toe off events at a given time $t_{TO}$ were identified as the valleys of the toe markers $x_{toe}$, also in the anterio-posterior direction. In addition, the foot markers were normalized with respect to the sacrum marker $x_{sacrum}$, resulting in the signal being centered around the origin, as shown in Equations (8) and (9):

(8) $$t_{HS}={\left(x_{heel}-x_{sacrum}\right)}_{max}$$

(9) $$t_{TO}={\left(x_{toe}-x_{sacrum}\right)}_{min}$$

As thresholds for the peak detection algorithm, the mean value of the anterio-posterior marker signals were used. Since the Plug-in Gait marker model does not include a marker at the sacrum, the average position of the right and left posterior superior iliac markers (RPSI and LPSI) was calculated to represent a virtual sacrum marker. For the Kinect cameras, the Spine-Base and Pelvis markers were used for Kinect v2 and Azure Kinect, respectively.

Based on the identified gait events, the spatio-temporal gait parameters were calculated as proposed in the study by Eltoukhy et al. [31]. The description of the gait parameter calculation for the Vicon and Kinect systems is presented in Table 2. Since the timing of the gait events is crucial for the accuracy of the calculated spatio-temporal gait parameters, the frame differences of the identified peaks between the Kinect and Vicon signals were evaluated at 100 Hz.

### 3.7. Statistical Analysis

The first goal of our experiment was to investigate spatial agreement of the corresponding joint coordinates between both Kinect cameras relative to the Vicon reference system. In order to calculate the accuracy of the Kinect markers, the three different marker models have to be mapped. Using the approach proposed by Otte et al. [32], a subset of the Vicon retro-reflective markers was mapped to the corresponding markers of the respective Kinect skeleton. This was achieved either by assigning individual Vicon markers that were closest to the corresponding Kinect landmark or, if several Vicon markers were present, by averaging the markers. Table A1 in the Appendix A presents the complete marker mapping in detail. Since the Vicon markers were applied to the participant’s skin while both Kinect cameras track the joints approximately within the user’s body (as shown in Figure 2a,b), an offset of the corresponding marker positions would cause a systematic bias when directly comparing the joint positions. In order to compare the joint trajectories in all three directions, this systematic bias was removed by subtracting the mean value of each axis of each joint [32]. These signals are referred to as zero-mean shifted signals, where similar movements should now have a high degree of agreement when moving in a certain direction.

After the marker models were mapped, the 3D Euclidean distance between the zero-mean shifted signals of corresponding joints were calculated over all *N* captured frames of all participants and trials and for each Kinect camera individually. The overall tracking accuracy was then assessed as mean and standard deviation of the Euclidean distances [32]. As this study included five participants, the non-parametric paired Wilcoxon test was performed using the mean values of the distances of all trials. Differences in tracking quality were found to be significant for *p*-values smaller than p<0.05 and p<0.01, respectively.

In addition to the paired Wilcoxon test, Pearson’s correlation coefficients were used to assess reliability [33]. The correlation was assessed of zero-mean shifted signals over all *N* captured frames of all subjects and trails. Pearson’s *r*-values were calculated for the three axes separately, in the following referred to as the anterio-posterior (AP), medio-lateral (ML), and vertical (V) axes [32,34]. The levels of agreement were set to poor (r<0.4), moderate (0.4≤r<0.7), good (0.7≤r<0.9) and excellent (r≥0.9) [32,35].

The agreement of the spatio-temporal gait parameters generated by the three camera systems was compared and evaluated over a range of individual treadmill velocities. Therefore, the average values of the Vicon gait parameters and the two Kinect sensors were calculated first.

Absolute $e_{a}$ (shown in Equation (10)) and relative errors $e_{r}$ (shown in Equation (11)) were assessed for each spatio-temporal gait parameter in order to obtain an average absolute magnitude of the differences between the systems as well as a directional magnitude of the differences [31]. Additionally, the root mean squared error (RMSE) between gait parameters calculated from the Vicon system and those of the Kinect sensors was determined:

(10) $$e_{a}=\left|Kinect-Vicon\right|$$

(11) $$e_{r}=Kinect-Vicon$$

To assess the statistical significance between the three camera systems, we performed the non-parametric paired Wilcoxon test on the the pooled data of five subjects by using the mean values of the according gait parameter of individual trials. A significant difference for gait parameters was defined for *p*-values with p<0.01.

## 4. Results

In this section, the evaluation of the motion tracking performance of the Azure Kinect sensor is presented and compared with the Kinect v2 camera with respect to the Vicon gold standard system. The evaluation is performed by utilizing the statistical metrics presented in Section 3.7 over all subjects and trials. It is important to note that one trial was excluded from the analysis as the laptop which was operating the Kinect v2 sensor was frozen for a short period of time during the recording session without notice.

### 4.1. Joint Position Agreement

The first subject of the evaluation was the spatial agreement between the systems when walking on the treadmill. Therefore, the position data were first segmented for the walking parts and then the 3D Euclidean distance between the joints was calculated. As shown in Figure 2b, most of the landmarks in the skeleton definition of Kinect v2 and Azure Kinect are located at the same position, but Azure Kinect introduces additional joints, which means they can not be mapped to the previous skeleton definition. Therefore, the clavicle joints were evaluated without a direct comparison with Kinect v2. With regard to the spinal joints, Azure Kinect provides a joint in the abdomen region, the so-called Spine Naval, where there is no counterpart in Kinect v2. Although the markers on the chest are named differently by the two Microsoft SDKs, they are considered to be the same joints in this study due to their proximity. As there were no Vicon markers present at fingertips and in the face, these Kinect markers were all omitted.

**Figure 6:**
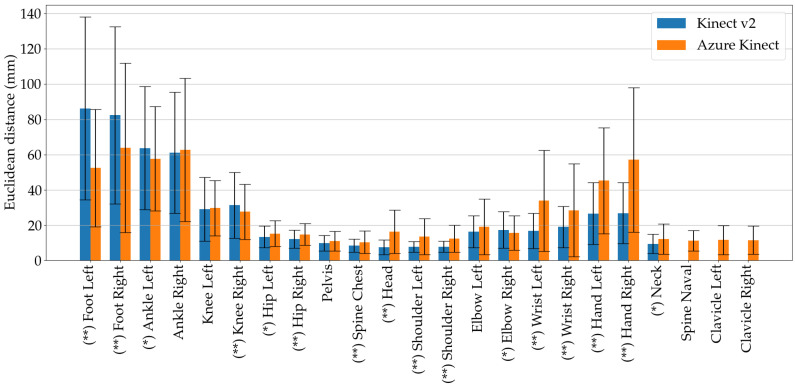
Spatial agreement of the Microsoft Kinect v2 and Azure Kinect cameras with respect to the Vicon system. Errors are represented as means and standard deviation of the 3D Euclidean distances between according joints.

**Figure A1:**
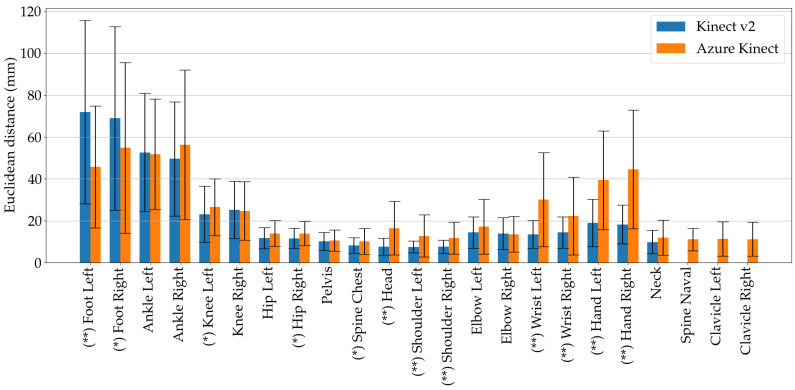
Spatial agreement between Microsoft Kinect v2 and Azure Kinect for 3.0 kmh${}^{-1}$.

**Figure A2:**
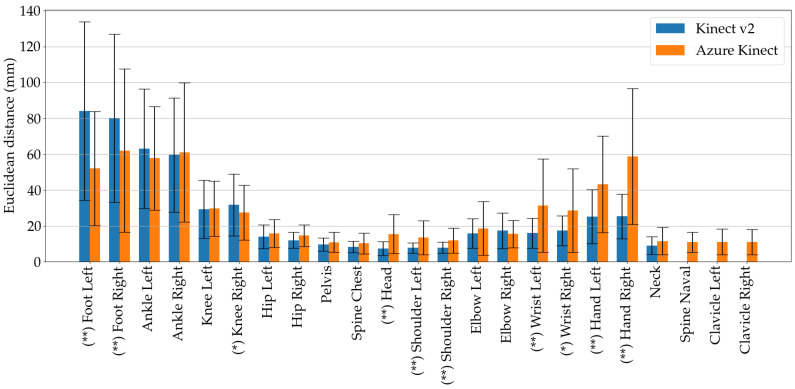
Spatial agreement between Microsoft Kinect v2 and Azure Kinect for 3.9 kmh${}^{-1}$.

**Figure A3:**
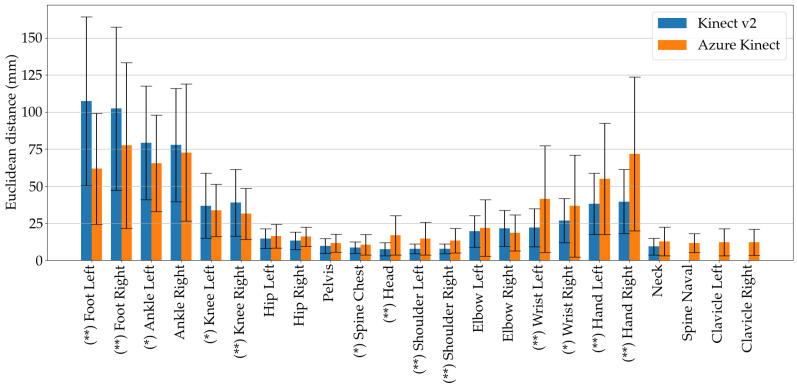
Spatial agreement between Microsoft Kinect v2 and Azure Kinect for 4.7 kmh${}^{-1}$.

The errors of the two Kinect sensors with respect to the Vicon ground truth system are presented in Figure 6, with the errors accumulated over all trials and provided as mean and standard deviation. In addition, the significant differences between each pair of joints were assessed using the non-parametric paired Wilcoxon test, where two levels of significance were defined and reported as (*) for p<0.05 and (**) for p<0.01. The assessment of spatial agreement for the three individual treadmill velocities is given in the Appendix A in Figure A1, Figure A2 and Figure A3.

Notably, the foot markers were tracked by the Azure Kinect sensor at all speeds with significantly higher accuracy. The Kinect v2 also showed a higher variance regarding the foot marker position. The differences of ankle markers between the systems were smaller, with only the markers on the left ankle showing a significant difference. However, the Kinect v2 camera showed better performance on upper body joints such as hip, spinal joints, head and shoulders. A higher error was found for both lower and upper extremities with both sensors, probably due to their faster movements and wider range of motion compared to, for example, hip joints.

In addition, Pearson correlation coefficient *r* for the anterio-posterior (AP), medio-lateral (ML) and vertical (V) directions were assessed. Pearson *r* values were calculated for each trial over all frames and the mean values and standard deviations were assessed. Table 3 shows that both Kinect cameras achieved excellent agreement for all joints in the AP direction. In the ML direction, Kinect v2 achieved more excellent *r* values than Azure Kinect. The lowest level of agreement was achieved in the V direction for both sensors. In particular, the *r* values showed a poor agreement for both foot markers for Kinect v2 with only r=0.11 and r=−0.01. These values improved for the Azure Kinect camera where the agreement ranged from moderate to good. The Azure Kinect also had only moderate and good agreement for the V direction, where Kinect v2 had three poor values.

### 4.2. Gait Event Timing

As explained in Section 3.6, the calculation of spatio-temporal gait parameters is based on heel strike and toe off events, which are automatically identified within the pose data. Given that the identification of these events is crucial for the calculation process, the identified gait events within the Kinect data were compared with those found within the Vicon signal. The errors of the gait event identification were expressed as differences of the frames between the identified peaks. To calculate the spatio-temporal gait parameters, the Kinect data were upsampled to 100 Hz in order to match the sampling frequency of the Vicon signal.

**Figure 7:**
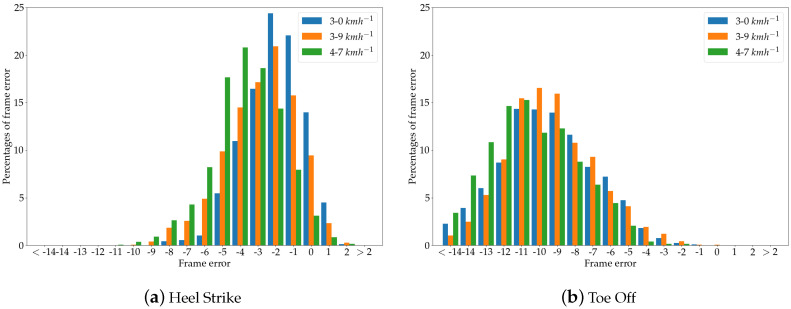
Gait event timing of Kinect v2 with respect to the Vicon reference system.

**Figure 8:**
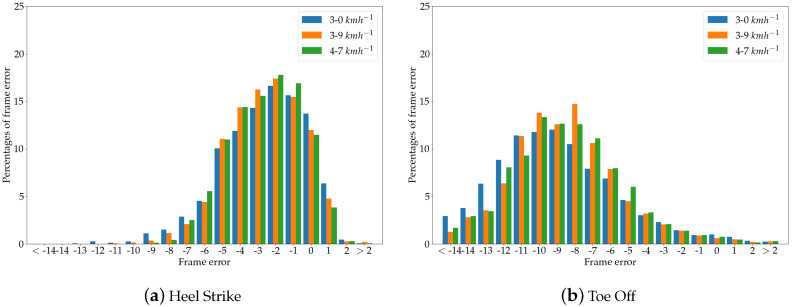
Gait event timing of Azure Kinect with respect to the Vicon reference system.

By analyzing the frame error of both gait events for each of the two Kinect cameras, it was shown that the detection of heel strike events generally has smaller errors compared to toe off events in both cameras. The Kinect v2 achieved a mean error of −2.8±2.0 frames, which is equal to −28±20 ms across all treadmill velocities, while the Azure Kinect camera had a mean error of −2.6±2.2 frames or −26±22 ms. The frame error distribution is shown in Figure 7a for the Kinect v2 camera, with Kinect gait events detected earlier for negative values and Kinect gait events detected later for positive values. The results for the Azure Kinect sensor are shown in Figure 8a.

In identifying toe off events, the Kinect v2 achieved a mean error of −9.8±2.7 frames or −98±27 ms, while the Azure Kinect sensor had a mean error of −8.8±3.4 frames or −88±34 ms. The distribution of errors for toe off events for each treadmill velocity are shown in the Figure 7b and Figure 8b for Kinect v2 and Azure Kinect, respectively.

The results presented here are in accordance with the study conducted by Xu et al. [23]. These authors also detected a smaller frame error in heel strike events. Overall, it was found that, for heel strike and toe off events identified within the upsampled Kinect data, the errors were within an acceptable range in order to calculate spatio-temporal gait parameters based on these gait events.

### 4.3. Spatio-Temporal Gait Parameters

**Figure 9:**
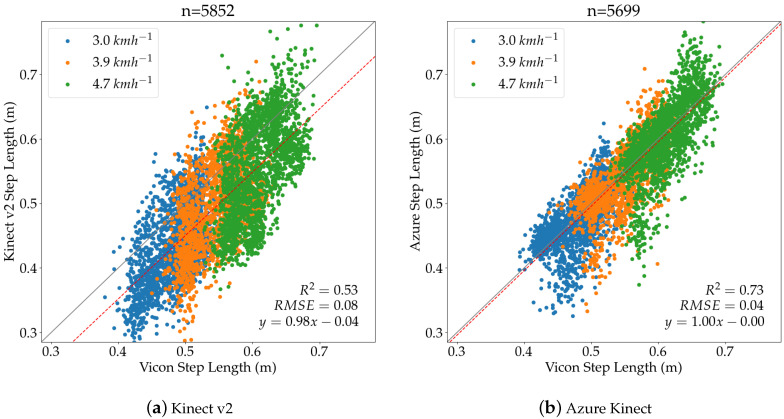
Step Length for Kinect v2 and Azure Kinect aggregated over all three speeds.

In this section, we present the evaluation of the spatio-temporal gait parameters of interest, i.e., step length, step width, step time, and stride time, using the metrics defined above. For step length and step width, additional correlation plots are provided for all treadmill speeds including a linear regression. The correlation plot for the step length parameter is shown in Figure 9a for Kinect v2 and in Figure 9b for the Azure Kinect, with the three colors representing the different treadmill speeds. The *x*-axes represent the calculated parameters coming from the Vicon system, while the *y*-axes represent the parameters calculated for each of the Kinect cameras. The graphs show that the three systems measure the increasing step length when increasing the treadmill speed. The regression line for the Azure Kinect camera is very close to the ideal line with having a slope of one and a small negative intercept. In contrast, the regression line of Kinect v2 has a larger offset, which is indicated by the negative intercept. The RMSE, calculated from the step lengths of Vicon and each of the Kinect cameras, confirms that the Azure Kinect sensor provided a higher accuracy compared to the previous Kinect model, measured over all treadmill velocities.

The evaluation of the step length parameter for individual treadmill velocities is shown in Table 4. It was found that the Azure Kinect camera performed better than Kinect v2 at all treadmill speeds, as indicated by the smaller absolute and relative error values, as well as the smaller RMSE values. The low *p*-values of the paired Wilcoxon test indicate a significant difference between the systems for all treadmill speeds.

**Figure 10:**
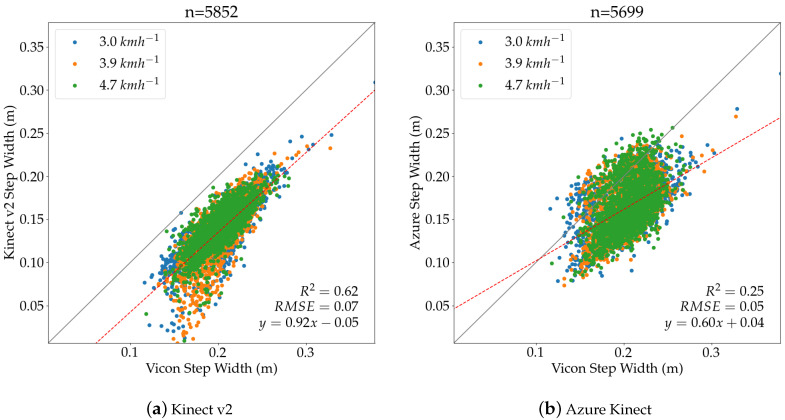
Step Width for Kinect v2 and Azure Kinect aggregated over all three speeds.

The evaluation of step width over all treadmill speeds is also shown as a correlation plot with a linear regression for the Kinect v2 camera in Figure 10a and for Azure Kinect in Figure 10b. It was found that, for the Azure Kinect, more measurements were closer to the ideal line, although the larger $R^{2}$ indicated a better fit of the measurements for Kinect v2. The smaller RMSE value confirms the increased accuracy in the Azure Kinect sensor.

Table 5 presents the results for step width and shows that the Azure Kinect camera outperformed the Kinect v2 camera at all treadmill velocities. It can be seen that the Azure Kinect achieved smaller absolute and relative mean errors and smaller RMSE values compared to its predecessor. For the speeds 3.0 kmh${}^{-1}$ and 4.7 kmh${}^{-1}$, the *p*-values were above the significance level, indicating a significant difference in the measured parameters.

For the step time parameter, it can be noticed that both Kinect camera generations performed very similar by delivering similar error values. Table 6 shows that, for the absolute error, the exact same values were achieved by both Kinect systems. For the relative error and the RMSE, both systems achieved almost similar values, except for Azure Kinect performing slightly better at 3.9 kmh${}^{-1}$. The *p*-values indicate that no significant differences were identified between the two systems.

The evaluation of stride time is illustrated in Table 7. It can be seen that, again, both Kinect sensors achieved similar absolute error values, but Azure Kinect delivered slightly better relative errors and RMSE values for all three treadmill velocities. The Wilcoxon test indicated no significant differences for this temporal gait parameter. In general, the estimated mean values from the Kinect sensors for the spatio-temporal gait parameters are comparable to those obtained in the study conducted by Xu et al. [23].

## 5. Discussion

In this study, the human pose estimation performance of the Azure Kinect camera was evaluated and compared to its predecessor model Kinect v2 in terms of gait analysis on a treadmill. The evaluation subject was the spatial agreement of joint locations as well as the quality of spatio-temporal gait parameters. A Vicon motion capturing system and the 39 marker full-body Plug-in Gait model were used to evaluate the parameters coming from both Kinect systems.

### 5.1. Results

The results of this study show that the tracking accuracy of the foot marker trajectories is significantly higher for the Azure Kinect camera over all treadmill velocities (3.0 kmh${}^{-1}$, 3.9 kmh${}^{-1}$, and 4.7 kmh${}^{-1}$) compared to the previous model Kinect v2. However, the Kinect v2 performed better than the Azure Kinect in the mid and upper body region, especially in the upper extremities. The newly added joints (clavicles, spine chest) of the Azure Kinect camera achieved a reasonable tracking error of about 11.5 mm. Regarding the gait parameters, it was shown that, for spatial parameters, i.e., step length and step width, the Azure Kinect camera delivered a significantly higher accuracy than Kinect v2. One possible reason for this could be the improved tracking quality of the foot markers of the Azure Kinect. However, for temporal gait parameters, i.e., step length and stride length, no significant differences were found between the two Kinect camera systems. Furthermore, the calibration method presented here is a tool that can be used in future similar studies for further evaluation of the cameras.

### 5.2. Study Limitations

Our results are specific to a population of young and healthy participants with normal, i.e., unpathological, gait patterns who walked at average walking speeds. The main objective of this pilot study was to demonstrate the technical feasibility of using the novel skeleton tracking algorithm of the Azure Kinect camera and to compare it with the previous model, Kinect v2. Therefore, no clinical research question was addressed in this study and a smaller sample size was regarded as sufficient.

Although a multi-camera motion capture system was considered the gold standard for evaluating human motion in this study, it is important to note that these systems have potential sources of error. The main drawback of retro-reflective marker-based systems, such as the Vicon system, is that each marker must be seen by at least two cameras at any time in order to be correctly interpolated. The study conducted by Merriaux et al. [36] examined the accuracy of the Vicon system for both static and dynamic setups with a 4-axis motor arm and a fast-moving rotor, respectively. These mechanical setups provided accurate ground truth data that was compared to the tracked Vicon data. They found that the Vicon system for the static mechanical setup had an average absolute positioning error of 0.15 mm and a variability of 0.015 mm, indicating high accuracy and precision. For fast dynamic movements of the markers, a mean position error of less than 2 mm was determined, whereas the variability in the static setup was lower.

Unlike passive tracking systems that use markers with reflective material, active marker systems use light emitting diodes (LED) that can be tracked by the individual cameras. These LEDs can be assigned to individual identifiers so that the system can easily identify markers after they have been lost, for instance due to occlusion. In passive marker systems, marker confusion in the tracking software is a common problem, especially if the markers move at higher speeds [37]. The confusion of markers can lead to more holes in the data and therefore requires manual effort to clean up the data in the software with interpolation strategies that introduce artificial artifacts into the data. In our study, we have used a 10-camera Vicon system and resulting holes in the data were filled using the Vicon Nexus software, which eventually introduced noise in the reference data.

With marker-based motion capture systems, the correct placement of active or passive markers on the subject is crucial for the quality of the kinematic data. The study conducted by Tsushima et al. [38] investigated the test–retest and inter-test reliability of a Vicon system. Kinematic data of the lower extremities were recorded using a repeated measurement protocol of two test sessions on two different days. Two trained experts placed 15 markers on the pelvis and lower body according to a predefined marker model. Across both testers and six unimpaired participants, the authors received high coefficients of multiple correlation (CMC) values for the sagittal plane ($R_{a}$ = 0.971–0.994), the frontal plane ($R_{a}$ = 0.759–0.977) and the transverse plane ($R_{a}$ = 0.729–0.899), excluding the pelvic tilt. From this, the authors conclude that the reduction of variability is possible if standardized marker placement methods are used.

In addition, soft tissue artifacts are a potential source of error in motion capture systems. These artifacts are caused by the movement of markers on the skin, which therefore no longer match the underlying anatomical bone landmarks. Lin et al. [39] quantified the effect of soft tissue artifacts in the posterior extremity of canines using a fluoroscopic computed tomography system with simultaneous acquisition of Vicon data using retro-reflective markers. It was found that the thigh markers and crus markers had a large peak amplitude of 27.4 mm and 28.7 mm, respectively. In addition, flexion angles were underestimated, but adduction and internal rotation were overestimated once the knee was flexed more than 90 degrees. Given this, it is likely that the recorded data in our study were also susceptible to soft tissue artifacts that led to more unstable data and results.

In gait analysis, the accurate detection of heel strike and toe off events is important as timing information is needed for the exact calculation of the temporal gait parameters and for the normalization of the data per gait cycle. The common gold standard method for the exact determination of these events are force plates [40]. O’Connor et al. [40] and Zeni et al. [30] presented algorithms for calculating these gait events based on kinematic data. However, the calculation of these events using motion capture data are flawed compared to force plate data. The so-called foot velocity algorithm for overground walking, as presented by O’Connor et al. [40], has an error of 16 ± 15 ms for heel strike and 9 ± 15 ms for toe off events and was verified on a data set containing gait of 54 normal children recorded with a force plate and a motion capture system. The method proposed by Zeni et al. [30] for detecting gait events on the treadmill was verified on a data set that included seven healthy and unimpaired participants as well as seven multiple sclerosis and four stroke patients. In healthy participants, the algorithm correctly identified 94% of gait events with a 16 ms error. In impaired participants, 89% of treadmill events were identified with a 33 ms error. Since we used the algorithm presented by Zeni et al. [30] for our study, it can be assumed that the error of their method is also reflected in our results, since we used the temporal gait parameters obtained from Vicon as a reference.

### 5.3. Practical Application

The experimental setup of this camera evaluation study presented here offers many advantages that could also be transferred to a real-life scenario. Since the two Kinect cameras do not require time-consuming placement of markers, systems using these cameras could be operated by individuals/patients living in remote or rural areas who do not have access to medical services. Findings from our study appear to be relevant for this type of tele medicine or tele rehabilitation approach, particularly for patients with neurological disorders, such as in stroke survivors who are in need of gait retraining. In addition, walking on a treadmill allows data recording even in homes with limited space, as well as recording a large amount of data in just one session. Von Schroeder et al. [41] have investigated how the gait of stroke patients can change over time. Therefore, data were collected from 49 stroke patients and 24 control subjects using a portable step analyzer (B & L Engineering, Santa Fe Springs, CA, USA). They found that the evaluated gait parameters improved over time, with the greatest change occurring within the first 12 months. With a home setup using tele medicine, similar to the setup shown in Figure 3a, which consists of only one treadmill and a single Kinect camera, it would be possible to record gait parameters that provide important insights into changes in gait performance. The algorithm described in Section 3.6 could serve as a basis to obtain more important gait parameters.

Besides the above presented concrete use case, such single-based Kinect camera systems could also be used in different environments, where the use of more complex systems, e.g., multi-camera 3D motion capturing systems, is not feasible. These include physiotherapy, rehabilitation clinics, field test conditions, and fitness centers.

### 5.4. Future Work

At the time of conducting this pilot study, the Azure Kinect Body Tracking SDK was in its most recent version (version 1.0.1.) and will most likely be subject to further changes in the future, which could lead to improvements in the tracking quality. At this stage, however, a pilot evaluation study was necessary to assess the tracking quality that can be achieved with the improved hardware and DL-based skeleton tracking algorithm to date.

Future research should validate that the Azure Kinect camera could also be used for measurements among different age groups and individuals with gait abnormalities (Parkinsonian gait, stroke, etc.), using a larger number of participants. Various aspects of human movement such as body weight exercises or overground walking should also be further investigated with the Azure Kinect camera.

## Figures and Tables

**Table 1:** Hardware comparison between Kinect v2 and Azure Kinect.

Property	Kinect v2	Azure Kinect
RGB Camera Resolution	1920 × 1080 px	3840 × 2160 px
IR Camera Resolution	512 × 424 px	1024 × 1024 px
Framerate	30 fps	30 fps
Field of View IR Camera	70 × 60 degrees	75 × 65/120 × 120 degrees
Field of View RGB Camera	84 × 53 degrees	90 × 59/90 × 74 degrees

**Table 2:** Calculation of spatio-temporal gait parameters for Vicon and both Kinect sensors. LANK and RANK stand for the left and right ankle markers [31].

Parameter	Vicon Calculation	Kinect Calculation
Step Length	Distance between LANK and RANKmarkers during heel strike event	Distance between both ankle jointsduring heel strike event
Step Time	Time between consecutive heel strikesof left and right foot	Same as Vicon
Step Width	Distance along the medio-lateral axisbetween LANK and RANK markers	Distance along the medio-lateral axisbetween left and right ankle markers
Stride Time	Time between two consecutive heelstrikes of the same foot	Same as Vicon

**Table 3:** Pearson’s correlation coefficient *r* in the AP, ML, and V direction. The last three joints are only present in the Azure Kinect skeleton definition and thus marked as not present (n.p.) for Kinect v2.

	Kinect v2			Azure Kinect		
	AP	ML	V	AP	ML	V
Foot Left	0.93±0.03	0.80±0.09	0.11±0.11	0.97±0.01	0.72±0.10	0.71±0.10
Foot Right	0.94±0.02	0.82±0.12	−0.01±0.11	0.95±0.03	0.76±0.07	0.69±0.10
Ankle Left	0.97±0.02	0.95±0.03	0.76±0.14	0.97±0.01	0.85±0.06	0.89±0.04
Ankle Right	0.97±0.02	0.97±0.01	0.78±0.09	0.96±0.02	0.81±0.06	0.84±0.06
Knee Left	0.98±0.02	0.95±0.02	0.41±0.21	0.97±0.01	0.87±0.04	0.74±0.06
Knee Right	0.98±0.02	0.93±0.03	0.35±0.20	0.98±0.01	0.94±0.03	0.73±0.13
Hip Left	0.98±0.02	0.95±0.02	0.80±0.13	0.99±0.01	0.89±0.05	0.60±0.17
Hip Right	0.98±0.01	0.96±0.02	0.78±0.11	0.98±0.02	0.91±0.05	0.68±0.09
Pelvis	0.99±0.00	0.94±0.03	0.86±0.10	0.99±0.01	0.96±0.02	0.67±0.12
Spine Chest	0.99±0.01	0.98±0.01	0.88±0.07	1.00±0.00	0.96±0.02	0.75±0.13
Head	0.99±0.01	0.99±0.01	0.94±0.03	0.96±0.06	0.96±0.02	0.62±0.15
Shoulder Left	0.99±0.01	0.99±0.01	0.92±0.04	0.99±0.01	0.97±0.01	0.66±0.11
Shoulder Right	0.99±0.01	0.99±0.01	0.92±0.05	0.99±0.01	0.97±0.02	0.63±0.19
Elbow Left	0.99±0.01	0.99±0.01	0.62±0.22	0.97±0.02	0.89±0.14	0.62±0.13
Elbow Right	0.99±0.00	0.98±0.01	0.50±0.22	0.99±0.01	0.95±0.05	0.64±0.18
Wrist Left	0.99±0.01	0.98±0.01	0.96±0.01	0.95±0.05	0.79±0.19	0.70±0.22
Wrist Right	0.99±0.01	0.97±0.01	0.95±0.03	0.97±0.03	0.81±0.09	0.73±0.15
Hand Left	0.98±0.01	0.97±0.02	0.95±0.03	0.95±0.04	0.76±0.12	0.68±0.24
Hand Right	0.98±0.01	0.97±0.02	0.94±0.04	0.91±0.07	0.68±0.17	0.58±0.20
Neck	1.00±0.01	0.98±0.01	0.65±0.27	0.99±0.01	0.97±0.02	0.70±0.13
Spine Naval	n.p.	n.p.	n.p.	0.99±0.01	0.97±0.01	0.70±0.13
Clavicle Left	n.p.	n.p.	n.p.	0.99±0.01	0.97±0.02	0.74±0.11
Clavicle Right	n.p.	n.p.	n.p.	0.99±0.01	0.97±0.02	0.75±0.13

**Table 4:** Step length gait parameter evaluation.

		3.0 kmh${}^{-1}$	3.9 kmh${}^{-1}$	4.7 kmh${}^{-1}$
Vicon Mean (m)	Kinect v2	0.48±0.03	0.55±0.04	0.61±0.03
	Azure Kinect	0.48±0.03	0.55±0.04	0.61±0.03
Kinect Mean (m)	Kinect v2	0.44±0.06	0.50±0.07	0.55±0.08
	Azure Kinect	0.48±0.05	0.55±0.05	0.60±0.06
Absolute Error (m)	Kinect v2	0.06±0.03	0.06±0.04	0.07±0.05
	Azure Kinect	0.03±0.02	0.03±0.02	0.03±0.03
Relative Error (m)	Kinect v2	−0.05±0.05	−0.05±0.06	−0.05±0.06
	Azure Kinect	0.00±0.03	0.00±0.04	0.01±0.04
RMSE	Kinect v2	0.07	0.08	0.08
	Azure Kinect	0.03	0.04	0.04
*p*-value		0.005	0.008	0.005

**Table 5:** Step width gait parameter evaluation.

		3.0 kmh${}^{-1}$	3.9 kmh${}^{-1}$	4.7 kmh${}^{-1}$
Vicon Mean (m)	Kinect v2	0.21±0.03	0.20±0.02	0.20±0.02
	Azure Kinect	0.21±0.03	0.20±0.02	0.21±0.02
Kinect Mean (m)	Kinect v2	0.14±0.03	0.14±0.03	0.14±0.02
	Azure Kinect	0.17±0.03	0.16±0.03	0.17±0.03
Absolute Error (m)	Kinect v2	0.07±0.02	0.07±0.02	0.06±0.02
	Azure Kinect	0.04±0.02	0.04±0.02	0.04±0.02
Relative Error (m)	Kinect v2	−0.07±0.02	−0.07±0.02	−0.06±0.02
	Azure Kinect	0.04±0.03	0.04±0.03	0.04±0.03
RMSE	Kinect v2	0.07	0.07	0.06
	Azure Kinect	0.05	0.05	0.05
*p*-value		0.005	0.011	0.005

**Table 6:** Step time gait parameter evaluation.

		3.0 kmh${}^{-1}$	3.9 kmh${}^{-1}$	4.7 kmh${}^{-1}$
Vicon Mean (s)	Kinect v2	0.64±0.04	0.56±0.03	0.52±0.03
	Azure Kinect	0.64±0.04	0.56±0.03	0.52±0.03
Kinect Mean (s)	Kinect v2	0.64±0.05	0.56±0.04	0.52±0.04
	Azure Kinect	0.64±0.04	0.56±0.04	0.52±0.04
Absolute Error (s)	Kinect v2	0.02±0.02	0.02±0.02	0.02±0.02
	Azure Kinect	0.02±0.02	0.02±0.02	0.02±0.02
Relative Error (s)	Kinect v2	0.00±0.03	0.00±0.03	0.00±0.03
	Azure Kinect	0.00±0.03	0.00±0.02	0.00±0.03
RMSE	Kinect v2	0.03	0.03	0.03
	Azure Kinect	0.03	0.02	0.03
*p*-value		0.959	0.066	0.721

**Table 7:** Stride time gait parameter evaluation.

		3.0 kmh${}^{-1}$	3.9 kmh${}^{-1}$	4.7 kmh${}^{-1}$
Vicon Mean (s)	Kinect v2	1.28±0.07	1.12±0.06	1.04±0.05
	Azure Kinect	1.28±0.07	1.13±0.06	1.04±0.05
Kinect Mean (s)	Kinect v2	1.28±0.07	1.12±0.07	1.04±0.06
	Azure Kinect	1.28±0.07	1.12±0.07	1.04±0.06
Absolute Error (s)	Kinect v2	0.02±0.02	0.02±0.02	0.02±0.02
	Azure Kinect	0.02±0.02	0.02±0.02	0.02±0.02
Relative Error (s)	Kinect v2	0.00±0.03	0.00±0.03	0.00±0.03
	Azure Kinect	0.00±0.02	0.00±0.02	0.00±0.02
RMSE	Kinect v2	0.03	0.03	0.03
	Azure Kinect	0.02	0.02	0.02
*p*-value		0.017	0.051	0.169

## Appendix A

**Table A1:** Skeleton mapping of the 25 joints Kinect v2 and 32 joints Azure Kinect model to the 39 marker Vicon Plug-in Gait model.

Index	Kinect v2/Azure Kinect Joints	Vicon Markers to Kinect v2	Vicon Markers to Azure Kinect
1	Foot Left	LTOE	LTOE
2	Foot Right	RTOE	RTOE
3	Ankle Left	LANK	LANK
4	Ankle Right	RANK	RANK
5	Knee Left	LKNE	LKNE
6	Knee Right	RKNE	RKNE
7	Hip Left	LASI	LASI
8	Hip Right	RASI	RASI
9	Spine Base/Pelvis	RASI, LASI, LPSI, RPSI	RASI, LASI, LPSI, RPSI
10	Spine Middle/Spine Chest	CLAV + (RASI, LASI, LPSI, RPSI)	STRN
11	Spine Shoulder/Spine Naval	CLAV	T10
12	Head	RFHD, LFHD, RBHD, LBHD	RFHD, LFHD, RBHD, LBHD
13	Shoulder Left	LSHO	LSHO
14	Shoulder Right	RSHO	RSHO
15	Elbow Left	LEBL	LEBL
16	Elbow Right	REBL	REBL
17	Wrist Left	LWRA + LWRB	LWRA + LWRB
18	Wrist Right	RWRA + RWRB	RWRA + RWRB
19	Hand Left	LFIN	LFIN
20	Hand Right	RFIN	RFIN
21	Neck	C7	C7
22	Hand Tip Left	-	-
23	Hand Tip Right	-	-
24	Left Thumb	-	-
25	Right Thumb	-	-
26	Clavicle Right	n.p.	CLAV + LSHO
27	Clavicle Left	n.p.	CLAV + RSHO
28	Eye Left	n.p.	-
29	Eye Right	n.p.	-
30	Ear Left	n.p.	-
31	Ear Right	n.p.	-
32	Nose	n.p.	-
